# Neural subgraph counting on stream graphs via localized updates and monotonic learning

**DOI:** 10.1371/journal.pone.0334724

**Published:** 2025-10-23

**Authors:** Zhen Xie, Wenzhe Hou, Feiyang Wu, Hao Xu

**Affiliations:** Laboratory for Big Data and Decision, National University of Defense Technology, ChangSha, HuNan, China; National University of Sciences and Technology College of Electrical and Mechanical Engineering, PAKISTAN

## Abstract

Graphs are a representative type of fundamental data structures. They are capable of representing complex association relationships in diverse domains. For large-scale graph processing, the stream graphs have become efficient tools to process dynamically evolving graph data. When processing stream graphs, the subgraph counting problem is a key technique, which faces significant computational challenges due to its #P-complete nature. This work introduces StreamSC, a novel framework that efficiently estimate subgraph counting results on stream graphs through two key innovations: (i) It’s the first learning-based framework to address the subgraph counting problem focused on stream graphs; and (ii) this framework addresses the challenges from dynamic changes of the data graph caused by the insertion or deletion of edges. Experiments on 5 real-word graphs show the priority of StreamSC on accuracy and efficiency.

## Introduction

Graph-structured data are widely used in representing complex relations in multiple fields [1]. Modern applications create a growing demand for the processing of dynamic data such as real-time social interactions [2] and evolving biological networks [3,4]. Stream graphs, where the edges and nodes are dynamically inserted to or deleted from the graphs, provide the capability of modeling such dynamic data. Subgraph counting is an essential task in stream graph analysis and management. It serves as a cornerstone for downstream tasks such as anomaly detection [5] and graph mining [6]. Subgraph counting on stream graphs aims to count the occurrences of subgraphs that are isomorphic to a given pattern (a.k.a. a query graph) in the stream graph at specific timestamps. Subgraph counting on stream graphs is a practically important task in various domains where interactions evolve over time and timely detection of structural patterns is essential. For example, in financial transaction networks, counting the emergence of subgraphs such as triangles or other special structures can help identify fraud rings or money laundering activities. In cybersecurity, subgraph counting on stream graphs enables the real-time detection of potential attack patterns, such as DDoS events, worm propagation, or anomalous communication structures. These applications demand real-time or near-real-time tracking of structural patterns, which static graph-based subgraph counting methods struggle to handle efficiently. Static methods typically recompute the count from scratch for each snapshot, leading to prohibitive overhead as the graph continuously evolves. In contrast, stream-graph-based methods aim to maintain counts incrementally as new edges arrive, making them far more suitable for continuous monitoring scenarios. Subgraph counting is a #P-complete problem [7], and solving it incurs prohibitively high computational costs. Due to its computational complexity and broad applications, this problem has attracted significant research attention.

To address the computational inefficiency and to process complex queries in stream graphs, we propose a deep learning framework for approximate subgraph counting. While existing learning-based methods achieve high accuracy on static graphs, they exhibit critical limitations when applied to streaming scenarios: (1) prohibitive latency under large-scale edge updates, (2) low sensitivity to fine-grained edge modifications, (3) ignorance of monotonic properties in the dynamic updates.

**Challenge 1**: Current methods (e.g., NSIC [8], NeurSC [9]) perform pair-wised matching between data and queries, which becomes slow for dynamically updating stream graphs due to redundant reprocessing.

**Challenge 2**: The changes caused by single-edge updates are slight for existing neural network methods to distinguish due to the over-smoothing nature of GNNs.

**Challenge 3**: Subgraph counts in stream graphs obey monotonicity: they monotonically increase with edge additions and decrease with deletions. In contrast, static graph methods inherently fail to preserve this property.

The above-mentioned three challenges render the existing learning-based subgraph counting methods on static graphs hard to process stream graphs. Motivated by these challenges and inspired by the principles of Incremental View Maintenance (IVM) [10] in traditional databases, we propose StreamSC which is specially designed for stream graphs. The key ideas of our approach are: Performing re-computation only on the affected parts of data graph during decomposition and nforcing model monotonicity through innovative loss function during training. The former identifies the parts affected by edge updates instead of performing full-graph reprocessing, which can reduce the scope of recomputation. The reduction in the scope of recomputation can not only decrease the computational cost (Challenge 1) but also relatively highlight the subtle changes in edges (Challenge 2). The latter employs a loss function designed for monotonicity. This allows the model to tend to increase its output when an edge is inserted and to decrease its output when an edge is deleted (Challenge 3). StreamSC basically follows LearnSC [7] to implement subgraph counting on the initial graph, and then propose (i) a fast re-decomposition mechanism to efficiently process updates in the stream graph and (ii) a method to capture the monotonicity features by learning the directed changes in updates. Our contributions are as follows:

- We propose StreamSC, an efficient learning-based framework, to predict subgraph counts on stream graphs. Our method explicitly tackles the limitations of static graph approaches when applied to dynamic streams, including redundant computation under updates and insensitivity to incremental changes.
- We design novel loss functions that preserve monotonicity under dynamic edge updates in stream graphs. It enforces the model to maintain consistency between subgraph count predictions and the inherent monotonic properties of stream graph updates.
- We propose an optimized data graph decomposition framework that accelerates processing through localized updates. By dynamically assessing the impact scope of edge modifications via incremental analysis, our method selectively recomputes only affected subgraphs. This approach, combined with historical data caching, eliminates redundant computations inherent to full-graph reprocessing.

The paper is structured as follows: Sect 1 formally defines the subgraph counting problem on stream graphs; Sect 2 reviews related work; Sect 3 presents our proposed method, StreamSC; Sect 4 evaluates StreamSC through experiments; Sect 5 discusses implications and limitations.

## 1 Problem definitions

In this section, we will give basic definitions of subgraph counting problem on stream graphs. The subgraph counting problem takes as input data graphs and query graphs. For certain query graphs, subgraph matching functions can map the query graphs to specific subgraphs within the data graph.

**Definition 1 (Subgraph matching function).** *For a given connected query graph* $Q(V_{Q},E_{Q})$*, data graph* $G(V_{G},E_{G})$ *and label function* L(·)*, if an injective function* $\phi :V_{Q}\to V_{G}$ *satisfies the following conditions, it is referred to as a subgraph matching function:*

*1.* $\forall u_{i}\in V_{Q},\phi (u_{i})\in V_{G}$ *and* $L(u_{i})=L(\phi (u_{i}))$*; and*
*2.* $\forall (u_{i},u_{j})\in E_{Q},\exists e\in E_{G}$ *that e connect* $\phi (u_{i})$ *and* $\phi (u_{j})$.

Intuitively, a subgraph matching function maps the nodes of a query graph to nodes in the data graph while preserving the adjacency relationships present in the query graph. Based on the subgraph matching function, we provide the definition of the subgraph counting problem.

**Definition 2 (Subgraph counting problem).** *For a given connected query graph* $Q(V_{Q},E_{Q})$, *connected data graph* $G(V_{G},E_{G})$ *and label function* L(·)*, subgraph counting problem is to compute the number of all possible different subgraph matching functions.*

It is worth noting that we restrict the query graph to be connected in our definition. For disconnected query graphs, their different connected components can be treated separately as individual query graphs. Our work focuses on the subgraph counting problem in stream graphs. Stream graphs are a type of dynamic graph representation where edges can be added or removed over time. Compared to static graphs, stream graphs have their own characteristics. To better define stream graphs, we first introduce the definition of stream graph record.

**Definition 3 (Stream graph record).** *A stream graph record can be represented by a triplet* r=(t,e,A) *where t is the timestamp when the record is assigned by the data source, e indicates an edge of the data graph, and A denotes the insertion or deletion of the edge e.*

Next, we provide the definition of stream graphs.

**Definition 4 (Stream graphs).** *A stream graph can be defined as an unbounded sequence of stream graph records* $S=\langle r^{1},r^{2},...\rangle$ *where each record* $r^{\text{m}}$ *arrives at a specific time* $t^{\text{m}}$.

**Fig 1:**
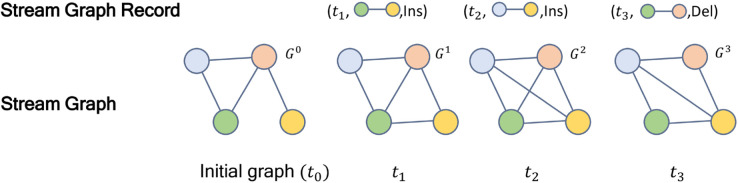
Example of stream graph.

As shown in Fig 1, the stream graph record captures the addition or deletion of edges at each moment after time *t*_0_, and based on the record, we can obtain the graph *G*_*t*_ at each moment. It should be noted that multiple edges may be added or deleted at each moment.

Next, we present the definition of the subgraph counting problem in stream graphs.

**Definition 5 (Subgraph counting on stream graphs).** *At the current time* $t^{\text{m}}$*, as illustrated in the* Fig 1*, a stream graph corresponds to a static graph* $G^{\text{m}}$ *at time* $t^{\text{m}}$*. The subgraph counting problem on stream graphs involves computing the subgraph count of a given query graph on* $G^{\text{m}}$*. For simplicity, we denote* $\text{c}(\text{Q},{\text{G}}^{\text{m}})$ *as the subgraph counting result for query graph Q in* $G^{\text{m}}$.

It should be emphasized that, although at time *t*_*i*_, the subgraph counting problem on stream graphs is equivalent to that on static graphs, the frequent changes in edges on stream graphs make neither the exact algorithms for static graphs nor retraining a learning-based model at each time step feasible.

Finally, we present a concept that is employed in the algorithm.

**Definition 6 (Edge match).** *Given an edge* $e(u_{i},u_{j})$*, and a graph* $G(V_{G},E_{G})$*, if* $\exists e(v_{i},v_{j})\in E_{G},L(u_{i})=L(v_{i})\wedge L(u_{j})=L(v_{j})$*, we define edge* $e(u_{i},u_{j})$ *can be matched to graph G, where the function* L(·) *maps each node to a specific label.*

## 2 Related work

In existing studies, although related methods exist, none offer a general solution for subgraph counting on stream graphs. There are indeed studies in related fields, however, they exhibit certain limitations when applied to stream graphs. We will introduce three main related fields, which are: subgraph counting on static graphs, subgraph counting of specific structures on stream graphs, and continuous subgraph matching.

### 2.1 Subgraph counting on static graphs

The subgraph counting methods on static graphs can be divided into two major categories: exact algorithms and approximate algorithms. The main exact algorithms currently available are Ullmann [11], VF2 [12], VF3 [13], and GraphQL [14]. Exact algorithms, in order to obtain accurate subgraph counting results, need to construct search trees for traversal and searching. Through continuous optimization of pruning methods, exact algorithms have made significant progress in terms of computational time. However, due to the inherent complexity of the subgraph counting problem, the time cost of exact algorithms still fail to meet the needs of subgraph counting on stream graphs.

The approximate strategies generally fall into two categories: statistical techniques and machine learning approaches. Early works were primarily based on statistical methods, which employ either graph sampling techniques or data summarization through sketch structures [15–18]. Recently, studies use deep learning techniques to address subgraph counting problem. NSIC [8] is the first to use a GNN model to estimate the count of a query graph. LSS [19] and NeurSC [9] were proposed to achieve more accurate predictions. LearnSC [7] proposes a unified framework for learning-based methods. Although learning-based subgraph counting methods have demonstrated good performance on static graphs, they require the entire query graph and data graph to be input into complex network structures. This leads to the repeated execution of graph neural network-related computations on large-scale data graphs when processing stream graphs. As a result, the existing approximate computing methods on static graphs also incur significant time cost when applied to stream graphs.

When dealing with dynamically evolving edges in stream graphs, static subgraph counting methods can only treat each snapshot as an independent static graph instance. This implies that, for *n* changes in the edge set (i.e., *n* snapshots), these methods must solve *n* separate subgraph counting problems. In practical scenarios, *n* is often large, leading to prohibitively high computational costs. For exact algorithms designed for static graphs, even a single subgraph counting task can be computationally intensive, making them impractical for stream graph settings. Although neural network-based methods for static graphs incur relatively lower overhead per snapshot, they are not optimized for the stream graph scenario. As a result, when *n* is large, their cumulative runtime can even exceed that of the exact continuous subgraph matching methods discussed later. Our comparative experiments on time overhead demonstrate this point. Since these methods are approximate rather than exact algorithms and do not show clear efficiency benefits in our setting. For this reason, such methods are not included as baselines in our subsequent experiments.

### 2.2 Subgraph counting on stream graphs

Significant progress has been made in counting specific subgraph structures in stream graphs. Several existing studies have been proposed in the literature [20–26]. For instance, PartitionCT [25] is a method specifically designed for counting the number of triangles in stream graphs. It leverages efficient graph partitioning and parallel processing techniques to rapidly and accurately count triangles in large-scale stream graphs. This method fully exploits the structural characteristics of the graph by dividing it into multiple subgraphs and performing triangle counting tasks in parallel on these subgraphs, thereby significantly improving computational efficiency. Furthermore, sGrapp [26] is another subgraph counting method for stream graphs, focusing on counting butterfly subgraphs (i.e., (2,2)-bipartite graphs). sGrapp employs an approximate algorithm based on adaptive windows, which can effectively handle the problem of counting butterfly subgraphs in large-scale stream graphs while maintaining high accuracy. This method optimizes algorithm performance by analyzing the temporal organization principles of butterfly subgraphs in stream graphs, enabling it to run efficiently on different stream graph data. These methods rely on traditional search-based techniques and are thus limited to handling only a specific query graph structure. As a result, they are not applicable to the subgraph counting problem as defined in our work. In contrast, our approach leverages a GNN-based framework to overcome this limitation.

Besides, some research has focused on multi-pass algorithms [27,28] for counting triangles and other subgraphs in graph streams. However, these algorithms need to traverse the graph stream multiple times to incrementally construct statistical information about subgraphs. This is essentially different from the subgraph counting problem on stream graphs addressed in this paper, as we cannot obtain the information of edges that will change subsequently.

### 2.3 Continous subgraph matching

Continuous Subgraph Matching (CSM) refers to the real-time detection of matching instances of a given query graph within a dynamic graph stream. As the graph data is continuously updated (such as through edge insertions and deletions), CSM algorithms are capable of efficiently identifying subgraph instances that satisfy the structure of the query graph. The CSM problem has been extensively studied in previous work [29–34]. Representative works in this area include GraphFlow [33] and RapidFlow [34]. GraphFlow is an active graph database system that optimizes the processing order of query nodes to reduce the generation of intermediate results, thereby accelerating the computation process. RapidFlow introduces a query reduction technique that transforms the continuous subgraph matching problem into a batch subgraph matching problem, and it handles graph streams by optimizing the matching order and leveraging efficient batch subgraph matching methods.

Through continuous subgraph matching, we can obtain precise subgraph counting results on stream graphs. Although these works have made some progress in reducing time cost, similar to exact static graph subgraph counting methods, the time cost of CSM methods remains unacceptable due to the inherent complexity of the subgraph matching problem.

## 3 StreamSC

To address the aforementioned challenges, we propose StreamSC, a deep learning subgraph counting framework with optimizations for stream graphs. StreamSC is inspired by IVM, as both share the core idea of replacing full recomputation with localized updates when handling changes. The main difference lies in their application domains: IVM is designed for traditional relational databases with well-structured data, whereas StreamSC targets graph data, which is inherently more complex in structure.

Specifically, it (i) utilizes an optimized data graph decomposition method that only processes the key part for each stream record, and (ii) captures latent monotonicity relationships of the changing graph topology to improve the estimation accuracy on stream graphs.

### 3.1 Framework

**Fig 2:**
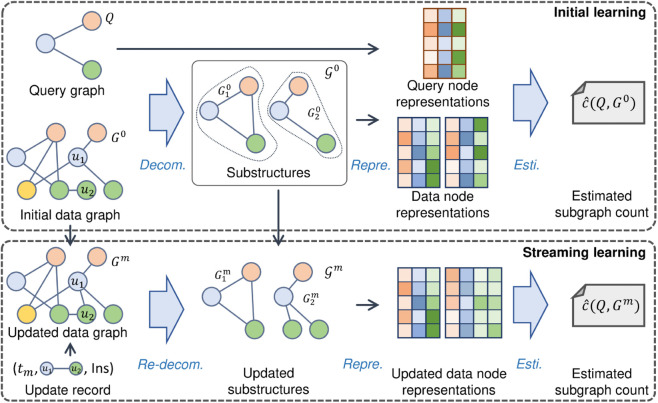
Framework of StreamSC.

As shown in Fig 2, StreamSC utilizes two stages to estimate the subgraph counts. The first stage is known as *initial learning*. It learns an estimation model on the initial data graph *G*^0^ and estimates the subgraph count for the input query graph *Q*. The second stage is *streaming learning*. When an update record comes, StreamSC quickly processes it in this stage.

Before introducing the core idea of streaming learning stage, we first outline the procedures of the initial learning stage, as this provides essential context for understanding StreamSC. As the initial graph *G*^0^ can be seen as a static graph, we follow the state-of-the-art subgraph counting method on static graphs, LearnSC [7], to train the initial model.

We now review the procedures of LearnSC with simplification because not all the phases in LearnSC are the focus in this work. LearnSC consists of five phases, namely decomposition, representation, interaction, estimation, and aggregation. Given a query graph *Q* and a data graph *G*^0^, (i) the decomposition phase selects the key nodes of the data graph and extracts critical substructures based on the selected nodes; (ii) the representation phase represent the nodes in the query graph and the substructures into vectors; (iii) the interaction, estimation, and aggregation phases use the representations vectors to produce a scalar output, which is known as the estimated subgraph count for *Q* in *G*^0^. Please note that, since the techniques in the interaction, estimation, and aggregation phases are not the focus in our work, we use “estimation phase” to denote the three connected phases in our work. This phase is based on neural networks, inputting representation vectors of the nodes in query graph *Q* and in the substructures ${𝒢}^{0}$, outputting a scalar as the estimated subgraph count $\hat{c}(Q,G^{0})$. It can be trained with an end-to-end training process.

In the streaming learning stage, the data graph keeps changing. This leads to the changing of the substructures. Thus, the model must fit the new substructures before giving accurate estimations. An intuitive way to compute the changing substructures is simply decomposing the snapshot *G^m^* at each timestamp *t^m^*. However, it is time-consuming with heavy redundant computations. For example, as shown in Fig 2, at timestamp *t^m^*, an edge connecting *u*_1_ and *u*_2_ is inserted, $G_{2}^{0}$ is updated into $G_{2}^{m}$, while $G_{1}^{0}$ remains unchanged ($G_{1}^{m}$ is the same as it).

Fortunately, the computation for $G_{1}^{0}$ is redundant. Thus StreamSC can skip this part and only collect the key parts to update substructures, which significantly reduces the computation. We will present the details in Sect 3.3.

Subsequently, due to the high parallelism and polynomial complexity of the estimation phase, we retain the structure of the estimation phase during the streaming learning stage.

Furthermore, the update records include insertions and deletions. They are naturally monotonic in the counts as we will elaborate in Sect 3.4. Thus we propose novel loss functions to capture the monotonicity features and improve the accuracy of dynamic estimations.

### 3.2 Initial learning

As mentioned in Sect 3.1, StreamSC trains a model with three phases (namely the decomposition, the representation, and the estimation phases) to estimate the subgraph counts.

#### Decomposition phase.

Given a query graph *Q* and an initial data graph *G*^0^, the decomposition phase excludes unqualified nodes in *G*^0^ and extracts substructures ${𝒢}^{0}=\{G_{1}^{0},G_{2}^{0},\ldots \}$ to reduce computation and improve the final estimation accuracy.

The node exclusion is based on the fact that not all data graph nodes have the chance to match to a query graph node. For example, if we assume a query graph whose nodes are all labeled red, then the nodes labeled green in a data graph will never match to any node in the query graph.

Methods in the literature [14,35,36] provide more comprehensive pruning strategies, and we refer to such methods as filters, among which GraphQL [14] is a representative filter.

Formally, given a query graph $Q(V_{Q},E_{Q})$ and a data graph *G*^0^(*V*^0^,*E*^0^), the filters maintain a candidate set ${𝒞}_{v_{i}}^{0}$ for each query graph node $v_{i}\in V_{Q}$. The candidate set ${𝒞}_{v_{i}}$ contains data graph nodes that have chance to match to the query node $v_{i}$.

We follow NeurSC [9] to select nodes in all candidate sets, and extract the connected components of the induced graph of the nodes from the data graph *G*^0^ as the substructures. Eqs (1) and (2) illustrate the derivation of substructures.

$${𝒞}^{0}=\underset{v_{i}\in V_{Q}}{⋃}{𝒞}_{v_{i}}^{0},$$ (1)

$${𝒢}^{0}=\mathrm{CONNCOMP}(\mathrm{INDSUB}(G^{0},{𝒞}^{0})),$$ (2)

where $\mathrm{INDSUB}(G^{0},{𝒞}^{0})$ returns the induced subgraph of node set ${𝒞}^{0}$ in the graph *G*^0^, while CONNCOMP(G) returns the set of connected components of a graph *G*.

As an example shown in Fig 2, through the decomposition phase, *G*_0_ is converted into two substructures $G_{1}^{0}$ and $G_{2}^{0}$ (the parts circled by dotted lines).

#### Representation phase.

The representation phase represents the nodes in the query graph *Q* and the substructures ${𝒢}^{0}$ into vectors.

Each node is labeled with an integer attribute (which is distinguished by colors in our examples). We use one-hot encode [7] to encode these nodes, and then feed them into multi layer GNNs to capture structural information [7]. The output for each node is a fixed-length vector.

#### Estimation phase.

We finally use the node representation vectors to produce the estimated subgraph count for *Q* in *G*^0^. As we elaborated in Sect 3.1, this phase is not the focus in our work, we follow the procedures of LearnSC [7]. In brief, LearnSC derives graph-level representations for both the query and data graphs by aggregating their respective node embeddings. These representations are then concatenated and passed through a Multi-Layer Perceptron (MLP) to produce the final output. For details of the network architecture, we refer interested readers to the original LearnSC paper.

### 3.3 Streaming learning

To address the dynamic nature of stream graphs, StreamSC decomposes the data graph into multiple substructures, which serve as input to its neural network to capture useful local features. Specifically, despite significant structural changes in snapshots across different timestamps compared to the initial graph, the decomposed substructures retain a high probability of similarity or consistency with historical records. This stability arises because the decomposition process selects candidate nodes and extracts connected subgraphs based on the query graph’s characteristics, while the query graph itself remains unchanged.

In LearnSC, the data graph decomposition phase filters out unqualified data graph nodes, and extracts important substructures. However, as update records come from the stream, the data graph is modified and the decomposed results continuously change, which leads to heavy computations for re-decomposition.

StreamSC addresses this challenge by partially updating the decomposed substructures. It begins with the initial graph as the data foundation, employing the unsupervised training method to train the initial model. For each streaming update (e.g., a new edge insertion), only a few substructures are affected. StreamSC re-decomposes solely the affected part of the data graph.

**Fig 3:**
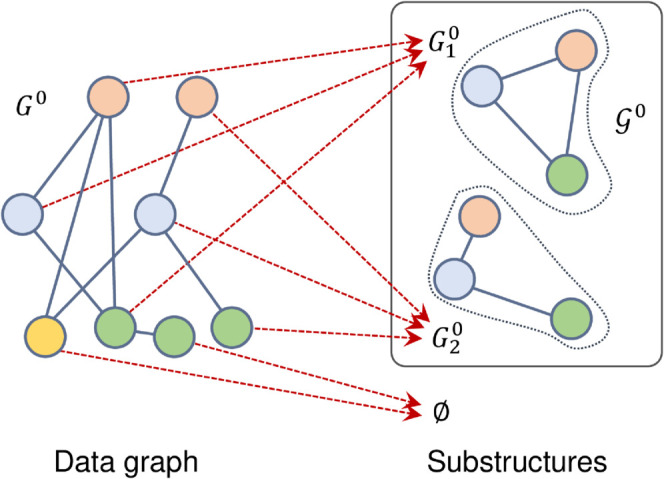
An example of the node anchor map.

To help rapid updates, we construct a node-to-substructure map, namely Node Anchor Map (NAM), as an auxiliary index, denoted as ${ℳ}^{m}:V_{G}^{m}\to {𝒢}^{m}\cup \{0\;\;\;/\}$, which maps data graph nodes $v^{m}\in V_{G}^{m}$ to the substructures $G_{i}^{m}\in {𝒢}^{m}$ to which they belong under the current timestamp. If a data graph node is not in any substructures, it is mapped to an empty set 0/. Fig 3 shows an example of the node anchor map. As shown in Fig 3, *G*^0^ is decomposed into ${𝒢}^{0}$. After decomposition, NAM is constructed by mapping the nodes in *G*^0^ to $G_{1}^{0}$, $G_{2}^{0}$ or 0/.

**Algorithm 1.** Updates



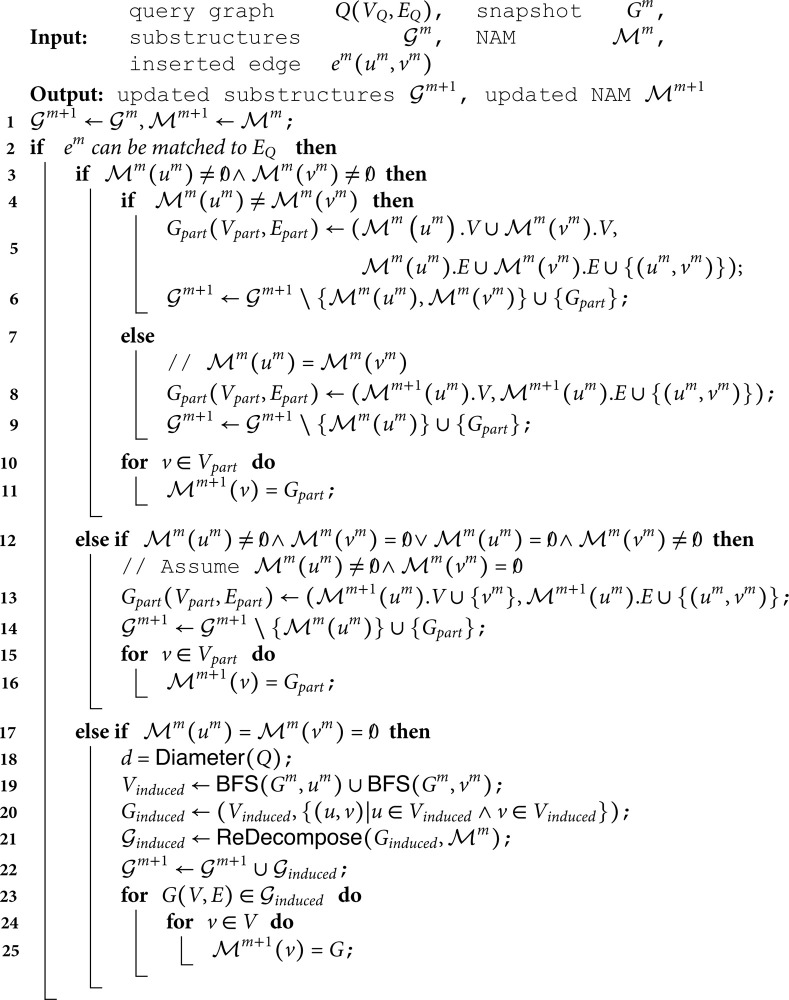



**Algorithm 2.** Redecompose



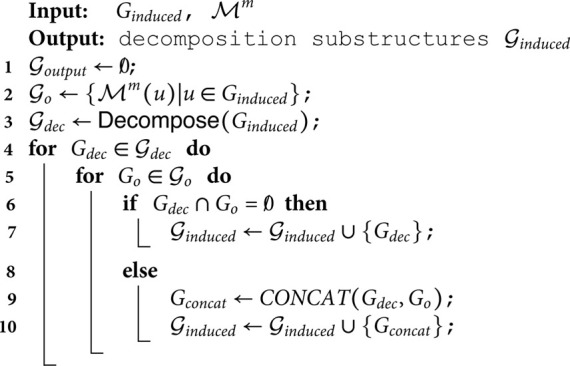



#### Insertion

Given a query graph *Q*, a data graph *G^m^* at timestamp *t^m^*, when a new edge $e^{m}=(u^{m},v^{m})$ arrives, Algorithm 1 lists the pseudocode of the updating procedures of the substructure set ${𝒢}^{m}$ and the NAM ${ℳ}^{m}$.

Algorithm 1 first checks whether the edge *e^m^* is matched to any edges of *Q* (Line 2). If not, the edge is abandoned as it will have no influence on (i) the substructures obtained in the decomposition phase or (ii) subgraph counting results. Otherwise, Algorithm 1 checks which part the nodes *u^m^* and *v^m^* belong to, and according to the specific scenarios, different update strategies are executed.

Specifically, we first analyze the underlying condition that (i) both *u^m^* and *v^m^* are already selected in the substructures (i.e., ${ℳ}^{m}(u^{m})\neq 0\;\;\;/\wedge {ℳ}^{m}(v^{m})\neq 0\;\;\;/$, Line 2). If they are not in the same substructure ${ℳ}^{m}(u^{m})\neq {ℳ}^{m}(v^{m})$, their corresponding substructures are concatenated with edge *e^m^* to obtain a new substructure *G*_*part*_ (see Line 5). Please note that we use ${ℳ}^{m}(\cdot ).V$ and ${ℳ}^{m}(\cdot ).E$ to denote the node and edge set of the substructure ${ℳ}^{m}(\cdot )$. Then the substructure set ${𝒢}^{m}$ removes the original substructures that *u^m^* and *v^m^* map to, and adds the new substructure *G*_*part*_, as shown in Line 6. This is based on the following observation. During the merge operation in Line 6, the edge (*u^m^*,*v^m^*) connects two original substructures. Line 5 connects these substructures into *G*_*part*_ through (*u^m^*,*v^m^*), preserving their local topologies. This is because the decomposition phase works based on the candidate filters, and neither the nodes nor edges in $ℳ(u^{m})$ and $ℳ(v^{m})$ transition from satisfying to violating the filtering criteria in Line 5. Thus, the merged *G*_*part*_ is inherently validated as a new substructure.

On the contrary, if *u^m^* and *v^m^* are in the same substructure (Line 7), the substructure is simply updated with inserting the edge *e^m^* as shown in Lines 8 to 9. Finally, we update the node anchor map for the influenced nodes (see Lines 10 to 10)

For another condition that (ii) only one of *u^m^* and *v^m^* is in a substructure. Due to the symmetric roles of *u^m^* and *v^m^*, we simplify the analysis by assuming that *u^m^* is in a substructure, while *v^m^* is not (i.e., ${ℳ}^{m}(u^{m})\neq 0\;\;\;/\wedge {ℳ}^{m}(v^{m})=0\;\;\;/$, Line 12). In this case, the substructure containing *u^m^* must be updated to contain *v^m^* at the next timestamp, because (i) *v^m^* connects to an available substructure and (ii) (*u^m^*,*v^m^*) can match to an edge in the query graph *Q*, thus it will not be filtered out. The substructure ${ℳ}^{m}(u^{m})$ is thereby updated by inserting node *v^m^* and edge (*u^m^*,*v^m^*) as shown in Lines 13 and 14.

Last, if (iii) neither of nodes *u^m^* and *v^m^* is in any substructures (i.e., ${ℳ}^{m}(u^{m})={ℳ}^{m}(v^{m})=0\;\;\;/$, Line 17), we extract the substructure that may contribute new matches as new edge inserted. In this case, we have Lemma 1.

**Lemma 1.** *Given a insertion edge e(*${\text{u}}^{\text{m}},{\text{v}}^{\text{m}}$*). If e(*${\text{u}}^{\text{m}},{\text{v}}^{\text{m}}$*) leads to new matches, then these new matches are necessarily contained in the induced subgraph with diameter r rooted at its endpoints(*${\text{u}}^{\text{m}},{\text{v}}^{\text{m}}$*). Where r is the diameter of query graph.*

This is because if the arrival of a insertion edge gives rise to new matches, it clearly implies that all such matches must contain the inserted edge. By extracting the subgraph around the newly inserted edge, we can ensure that all newly generated matches are covered. Specifically, StreamSC perform a breadth-first search with the nodes *u* and *v* as starting points, and the diameter of the query graph *Q* as the depth (see Line 18 to 19). A induced subgraph *G*_*induced*_ for the visited nodes are then extracted from the data graph *G* (see Line 20). *G*_*induced*_ and ${ℳ}^{m}$ is input into ReDecompose(·) function and we can get the new substructures ${𝒢}^{induced}$ (see Line 21). Details about the ReDecompose(·) function are listed in Algorithm 2. The substructures in ${𝒢}^{induced}$ are then added into ${𝒢}^{m+1}$.

Algorithm 2 demonstrates how StreamSC performs re-decomposition. First, Algorithm 2 utilizes NAM to obtain the set of substructures that overlap with *G*_*induced*_. It then decomposes *G*_*induced*_, as the procedures presented in the initial learning stage, to obtain its decomposition results ${𝒢}_{dec}$. Subsequently, Algorithm 2 iterates through ${𝒢}_{dec}$ and examines whether a particular decomposed part *G*_*dec*_ overlaps with any existing substructures *G*_*o*_. If *G*_*dec*_ does not overlap with any *G*_*o*_, it is directly merged into the final decomposition result set ${𝒢}_{output}$. If there is an overlap, *G*_*dec*_ is concatenated with all overlapping *G*_*o*_ to form a new graph *G*_*concat*_, which is then merged into the ${𝒢}_{output}$.

The function Decompose(·) in Algorithm 2 is consistent with the Equation (2). As shown in the Algorithm 1, only when ${ℳ}^{m}(u^{m})={ℳ}^{m}(v^{m})=0\;\;\;/$, Algorithm 2 is called to execute the time-consuming Equation (2). Furthermore, StreamSC selects only the necessary small part of the data graph in Algorithm 2 to reduce the computation. Consequently, StreamSC estimates the counting results much more efficiently than existing methods.

**Fig 4:**
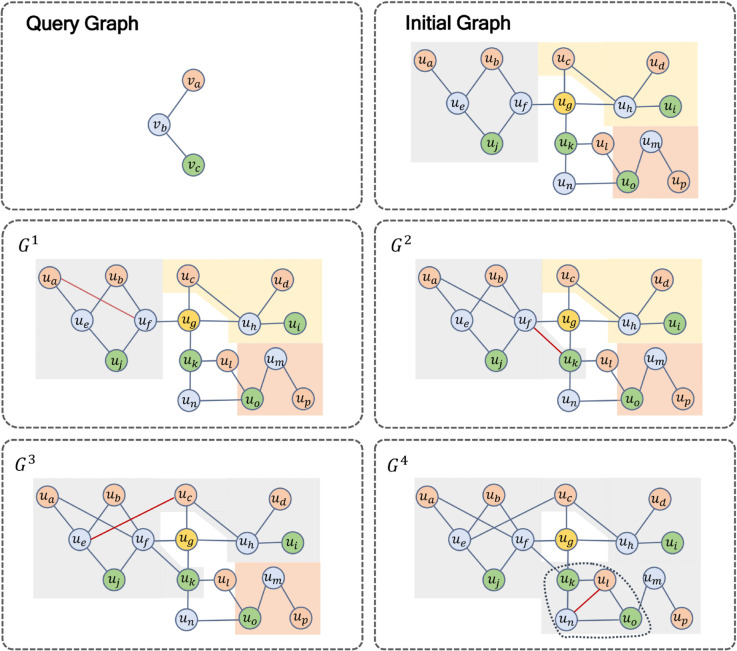
Examples of re-decomposition.

As shown in Fig 4, given a query graph and a data graph, the decomposition operation is performed in the initial graph. In this example, after decomposing the initial graph, the three subgraphs obtained (the areas covered in gray, yellow, and red in Fig 4 initial graph) represent the primary decomposition results. As time progresses, the edges in the stream graph undergo continuous changes.

- In Fig 4-*G*^1^, the newly arrived edge is $\left(u_{a},u_{f}\right)$. $\left(u_{a},u_{f}\right)$ connects nodes within the gray region. Only this region requires recomputation. Therefore, the gray region containing the new edge is directly fed into the model for recomputation, while the remaining parts remain unchanged.
- In Fig 4-*G*^2^, the newly arrived edge is $\left(u_{f},u_{k}\right)$ with *u*_*f*_ inside and *u*_*k*_ outside existing substructures. Thus, the gray region is extended to *u*_*k*_, and the updated gray region is then fed into the model, while the rest remains unchanged.
- In Fig 4-*G*^3^, the newly arrived edge is $\left(u_{e},u_{c}\right)$. $\left(u_{e},u_{c}\right)$ bridges two existing substructures. To maintain the connectivity of the substructures, the original gray and yellow regions are merged into a unified gray region, which is then fed into the model, while the other parts remain unchanged.
- In Fig 4-*G*^4^, the newly arrived edge is $\left(u_{i},u_{n}\right)$, with neither endpoints inside existing substructures. The diameter of query graph is 2. Thus, the algorithm extracts the 2-hop induced subgraphs starting from nodes *u*_*i*_ and *u*_*n*_. Consequently, the induced subgraph is then re-decomposed. The part circled by dotted line is the re-decomposition result. This part overlaps two existing substructures, so they are merged to a new substructure and the new substructure is fed the into model.

The example in Fig 4 intuitively demonstrates that our algorithm StreamSC can efficiently solve the subgraph counting problem in streaming graphs. By carefully designing the algorithm, StreamSC avoids unnecessary redundant computations while simultaneously reducing the size of input graphs for the neural network model, thereby achieving high processing efficiency.

#### Deletion

We subsequently present the re-decomposition phase for deletion operations. The procedures for a deletion are similar to those for an insertion. StreamSC selects a part of the snapshot that may lead to the change of substructures. Then we decompose the selected part to generate new substructures. Finally, we update the NAM index to keep the consistency.

Now we detail the selection of the part to be re-decomposed, while the updating process follows the Algorithm 1 for insertions. Given a query graph *Q*, as an edge $e^{m}=(u^{m},v^{m})$ is to be deleted, snapshot *G^m^* is updated to *G*^*m* + 1^. If (i) neither of nodes *u* and *v* appears in substructures ${𝒢}^{m}$, the deletion will not influence existing substructures, so the ${𝒢}^{m}$ and the NAM ${ℳ}^{m}$ do not need updates. Else if (ii) *u^m^* or *v^m^* is already selected in substructures (i.e., ${ℳ}^{m}(u^{m})\neq 0\;\;\;/\vee {ℳ}^{m}(v^{m})\neq 0\;\;\;/$), StreamSC concatenates the related substructures into a new graph *G*_*sub*_, and directly decompose *G*_*sub*_. Formally, $G_{sub}=\mathrm{CONCAT}({ℳ}^{m}(u^{m})\cup {ℳ}^{m}(v^{m}))$, where CONCAT(𝒢) simply concatenates the graphs $G_{i}(V_{i},E_{i})$ in set 𝒢:

$$\mathrm{CONCAT}(𝒢)=(\underset{1<i\leq |𝒢|}{⋃}V_{i},\underset{1<i\leq |𝒢|}{⋃}E_{i}).$$ (3)

StreamSC decomposees *G*_*sub*_ to generate new substructures, which replace the substructures in ${ℳ}^{m}(u^{m})\cup {ℳ}^{m}(v^{m})$. It then updates the maps in NAM ${ℳ}^{m}$ to the substructures to get ${ℳ}^{m+1}$ at the next timestamp *t*^*m* + 1^.

We present a concise justification that localized update is equivalent to re-decomposition from the global graph in the context of subgraph counting. Our reasoning is that, as long as the nodes matching the query graph remain within the updated substructures, localized update retains all critical information necessary for subgraph counting, and thus achieve functional equivalence to global re-decomposition from global graph.

- Given a new edge $\left(u_{m},v_{m}\right)$, if at least one of the nodes *u*_*m*_ or $v_{m}$ is already included in the existing substructure, the localized update proceeds by directly incorporating the new node(s) and the edge $\left(u_{m},v_{m}\right)$. Since the arrival of *u*_*m*_ and $v_{m}$ only modifies their immediate neighborhoods, any node *k* that was previously excluded from the substructure must have had a label or the number of neighboring nodes with specific labels that failed to meet the matching constraints of any query node. This condition remains unchanged upon the arrival of *u*_*m*_ and $v_{m}$. Therefore, simply adding *u*_*m*_, $v_{m}$, and $\left(u_{m},v_{m}\right)$ to the substructure is sufficient to preserve all the necessary information for accurate subgraph counting.
- In cases where both *u*_*m*_ and $v_{m}$ are outside the existing substructure, let the query graph have a diameter *r*, and define *G*_*sub*_ as the *r*-hop subgraph in the data graph centered at *u*_*m*_ and $v_{m}$. A potential difference between localized updates and re-decomposition from the global graph is that a node $k\in G_{sub}$ may be retained by the global re-decomposition but omitted by localized updates, as the latter considers only the local neighborhood of *k* within *G*_*sub*_. Nevertheless, if *k* is capable of matching a query node, then its neighborhood within *G*_*sub*_ must already satisfy the query’s structural constraints. This is because any new subgraph isomorphic to the query must be entirely contained within *G*_*sub*_. Consequently, although the results may differ from those obtained through re-decomposition from the global graph, executing a localized update guarantees that no critical information necessary for accurate subgraph counting is lost.
- In the case of edge deletion, any subgraph that can match the query graph and is subsequently removed must lie within the $G_{\text{sub}}$ defined by the algorithm. As discussed earlier, applying a localized update by performing decomposition on the resulting $G_{\text{sub}}$ ensures that no critical information necessary for subgraph counting is lost.

### 3.4 Monotonicity augmentation

In stream graphs, when edges are incrementally inserted over time, the values of subgraph counting satisfy the property of weak monotonicity: as a new edge comes, the subgraph counting result for a specific query graph only becomes larger or remains the same. The formal expression is illustrated in Lemma 2.

**Lemma 2.** *Given a query graph Q and a snapshot* ${\text{G}}^{\text{m}}$*, which is updated into G*^*m* + 1^ *by inserting an edge* ${\text{e}}^{\text{m}}$*, the subgraph counting value* $c(Q,G^{m+1})\geq c(Q,G^{m})$.

*Proof: G*^*m* + 1^ is obtained by inserting an edge into *G^m^*, so *G^m^* is a subgraph of *G*^*m* + 1^ and *G*^*m* + 1^ includes all nodes and edges of *G^m^*. Thus, based on Definition 1, all subgraph matching functions *ϕ* for *Q* in *G^m^* also hold in *G*^*m* + 1^, and the subgraph counting result (i.e., the number of available subgraph matching functions) for *Q* in *G*^*m* + 1^ is not less than that for *Q* in *G^m^*.

Similarly, we have Lemma 3 for updating by deleting an edge. We omit the proof here.

**Lemma 3.** *Given a query graph* Q *and a snapshot* ${\text{G}}^{\text{m}}$*, which is updated into G*^*m* + 1^ *by deleting an edge* ${\text{e}}^{\text{m}}$*, the subgraph counting value* $c(Q,G^{m+1})\leq c(Q,G^{m})$.

This monotonicity relation implicitly captures the dynamic evolving characteristics of stream graphs during updates: (i) it indicates that edge insertion (resp., deletion) produce increasing (resp., decreasing) results; and (ii) different edges may induce distinct magnitudes of result variation, reflecting their heterogeneous importance to specific queries. To model such dynamic trends, we design a novel loss function that (i) guides subgraph counting results to remain non-decreasing (resp., non-increasing) for insertions (resp., deletion) and (ii) captures residuals in subgraph counting results, emphasizing changes triggered by edges with significant residuals.

Let $\hat{r}=\hat{c}(Q,G^{m+1})-\hat{c}(Q,G^{m})$ (resp., $r=c(Q,G^{m+1})-c(Q,G^{m})$) denote the residual for estimated values (resp., true values), where $\hat{c}(\cdot ,\cdot )$ and c(·,·) denote the estimated subgraph counting values and the true values respectively.

We define the loss function for the tendency (i) as a marginal loss:

$${ℒ}_{oprt}(\hat{r})=\left\{ \begin{array}{cc} \max\{0,\tanh(-\hat{r})\} & \text{if }A=\text{Insertion},\\ \max\{0,\tanh(\hat{r})\} & \text{if }A=\text{Deletion}. \end{array}\right.$$ (4)

We use MSE to define the loss function for the tendency (ii).

$${ℒ}_{resd}(\hat{r},r)=(\hat{r}-r{)}^{2}/r_{max}^{2}.$$ (5)

Thus the final loss for the weak monotonicity property is defined as


$${ℒ}_{mono}={ℒ}_{oprt}+{ℒ}_{resd}.$$

### 3.5 Training

Based on the aforementioned loss function, we can derive the model training process. Since we cannot access information beyond the current time step in the subgraph counting problem on stream graphs, we are unable to directly compute the ${ℒ}_{mono}$ using *G*_*m* + 1_ during training. Therefore, StreamSC employs a sampling-based method to compute ${ℒ}_{mono}$. Specifically: given a initial data graph *G*^0^, we first randomly sample various query graphs from it. For each query graph, StreamSC firstly decomposes data *G*^0^ to get substructures ${𝒢}^{0}$. For every substructure $G_{i}^{0}$ the neural model estimates the counting result ${\hat{c}}_{i}(Q,G_{i}^{0})$, at the same time StreamSC randomly inserts/deletes edges from $G_{i}^{0}$ to get $G_{i}^{0+}$ and $G_{i}^{0-}$. $G_{i}^{0+}$ and $G_{i}^{0-}$ are fed into model to estimate the result ${\hat{c}}_{i+}(Q,G_{i}^{0+})$ and ${\hat{c}}_{i-}(Q,G_{i}^{0-})$.

We compute the regression loss for the estimated results:

$$\hat{c}=\sum_{i}{\hat{c}}_{i},$$ (6)

$${ℒ}_{reg}(\hat{c},c)=(log(\hat{c})-log(c){)}^{2},$$ (7)

where *c* represents *c*(*Q*,*G*^0^), denoting the true value. The ${ℒ}_{mono}$ is computed as:

$${\hat{r}}_{1}={\hat{c}}_{i}-{\hat{c}}_{i-},\;{\hat{r}}_{2}={\hat{c}}_{i+}-{\hat{c}}_{i},$$ (8)

$$r_{1}=c_{i}-c_{i-},\;r_{2}=c_{i+}-c_{i},$$ (9)

$${ℒ}_{mono}={ℒ}_{oprt}({\hat{r}}_{1})+{ℒ}_{oprt}({\hat{r}}_{2})+{ℒ}_{resd}({\hat{r}}_{1},r_{1})+{ℒ}_{resd}({\hat{r}}_{2},r_{2}),$$ (10)

where $c_{i-},c_{i},c_{i+}$ are true values computed by the exact subgraph counting method. We then combine the loss with the loss for weak monotonicity as

$$ℒ=\lambda_{reg}{ℒ}_{reg}+\lambda_{mono}{ℒ}_{mono}.$$ (11)

where $\lambda_{reg},\lambda_{mono}\in [0,1]$ are hyperparameters.

## 4 Experiments

In this section, we experimentally validate the accuracy and efficiency of StreamSC. The previously mentioned hyperparameters ($\lambda_{reg},\lambda_{mono}$) were tested on a small-scale dataset, and we found that setting them to 0.3 and 0.7 respectively is a reasonable choice. For more details, please refer to S2 Appendix. All the experiments are conducted on a Ubuntu 20.04 workstation with 64 Intel Xeon Gold 6346 @ 3.10GHz CPUs, NVIDIA Tesla A100 80GB GPU and 128GB RAM.

### 4.1 Datasets

We employed five datasets of varying sizes and different numbers of changed edges, commonly used in the field of subgraph counting, to conduct our experimental validation. All datasets used in this study are publicly available, and we have provided a convenient access link [37].The data collection and analysis methods in this study comply with the terms and conditions set forth by the dataset provider. Below is a brief introduction to these datasets.

- **Yeast** originates from the field of biology and represents a yeast protein-protein interaction network.
- **Citeseer** Citeseer is a well-known academic citation network dataset. Each paper is regarded as a node, and the citation relationships between papers form directed edges.
- **Wordnet** is a lexical semantic network dataset for the English language. Each word is represented as a node, and the edges between nodes signify the semantic relationships between words.
- **Wiki** is typically sourced from Wikipedia, an online encyclopedia based on collaborative editing by users. Each page serves as a node, and the hyperlinks between pages form directed edges.
- **Netflex** Netflix dataset originates from Netflix Inc., and comprises user ratings of movies. It constructs a network where users and movies are represented as nodes, and the ratings given by users to movies serve as the weights of the edges.

The statistics of the datasets are shown in Table 1.

**Table 1:** Statistics information of datasets.

Dataset	# of nodes	# of edges	# of nodes labels	Average Degree	# of changed edges
Yeast	3112	12519	71	8.046	1251
Citeseer	3264	4536	6	2.779	453
Wordnet	40559	71925	16	3.547	719
Wiki	5201	198353	14	76.275	1983
Netflex	3114895	2678693	37	1.719	2678

### 4.2 Baselines

StreamSC is the first learning-based method which focus on subgraph counting problem on stream graphs. To evaluate StreamSC, we implemented an exact algorithm and three approximate algorithms to validate our approach.

- **Exact methods**. The foundation of the subgraph counting problem is the subgraph matching problem. Therefore, we modified an exact algorithm RapidFlow [34] for subgraph matching on stream graphs to enable it to compute the exact results for the subgraph counting problem on stream graphs.
- **Approximate algorithms**. No approximation methods, whether based on statistics or artificial intelligence, have been proposed in previous work. In recent years, Graph Neural Networks (GNNs) have demonstrated remarkable performance across a wide range of tasks. We applied Graph Isomorphism Networks (GIN), Graph Attention Networks (GAT), and Long Short-Term Memory (LSTM) networks to the subgraph counting problem on stream graphs as baseline approximate computation methods.

### 4.3 Evaluation results

#### Accuracy of StreamSC.

We now compare StreamSC with approximate baselines to evaluate StreamSC’s performance. By comparing the q-error on different test sets and the variation of q-error with the increase of edges, we demonstrate the effectiveness of StreamSC. The definition of q-error is: $q\text{-error}(\hat{c},c)=\text{max}(\frac{\text{max}(1,\hat{c})}{\text{max}(1,c)},\frac{\text{max}(1,c)}{\text{max}(1,\hat{c})}).$ Naturally, we expect the subgraph counting method on stream graphs to maintain relatively stable performance even after substantial edge modifications. In other words, we aim to prevent the method’s error from exhibiting a significant increasing trend as edges change. Therefore, we conduct further analysis on the q-error sequences generated in the aforementioned experiments. We incorporate the Mann-Kendall (MK) test from time series analysis. The comparative analysis of *Z*_*mk*_ and corresponding *p*-values provides further validation of the methods’ performances. The MK test is a non-parametric statistical method widely employed to detect monotonic trends in time series or sequential data without assuming any specific data distribution. Below we formalize its computational procedure and key concepts:

For a sequential series $X=\{x_{1},x_{2},\ldots ,x_{n}\}$, the MK statistic *S* is computed as:

$$S=\sum_{i=1}^{n-1}\sum_{j=i+1}^{n}sgn(x_{j}-x_{i})$$ (12)

where the sign function sgn(·) is defined as:

$$sgn(x_{j}-x_{i})=\left\{ \begin{array}{cc} +1 & \text{if }x_{j}>x_{i}\\ 0 & \text{if }x_{j}=x_{i}\\ -1 & \text{if }x_{j}<x_{i} \end{array}\right.$$ (13)

The variance of *S* is defined as:

$$Var(S)=\frac{n(n-1)(2n+5)-\sum_{t}t(t-1)(2t+5)}{18}$$ (14)

Here, *t* is the count of ties for each repeated value. The standardized *Z*_*mk*_-score is calculated as:

$$Z_{mk}=\left\{ \begin{array}{cc} \frac{S-1}{\sqrt{Var(S)}} & \text{if }S>0\\ 0 & \text{if }S=0\\ \frac{S+1}{\sqrt{Var(S)}} & \text{if }S<0 \end{array}\right.$$ (15)

The statistical significance (*p*-value) corresponding to the computed *Z*_*mk*_-score is determined by evaluating its position in the standard normal distribution. For a given significance threshold *α* (set to 0.05 in this paper), a *p*-value less than *α* indicates the presence of a statistically significant trend in the sequence. Specifically:

- A positive *Z*_*mk*_ (*Z*_*mk*_> 0 ) denotes a significant increasing trend.
- A negative *Z*_*mk*_ (*Z*_*mk*_< 0 ) represents a significant decreasing trend.

Where the absolute magnitude of *Z*_*mk*_ reflects the strength of the detected trend. In the context of subgraph counting on stream graphs, q-error sequences demonstrating *p*-values above the threshold (p≥α) coupled with minimal absolute *Z*_*mk*_-values correspond to the most stable performance characteristics.

**Fig 5:**
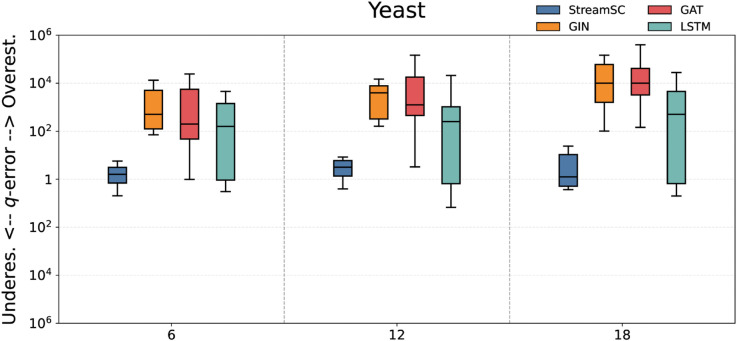
Subgraph counting results on Yeast.

**Fig 6:**
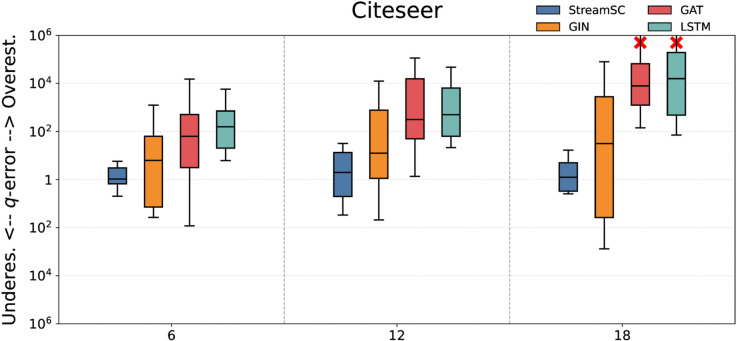
Subgraph counting results on Citeseer.

**Fig 7:**
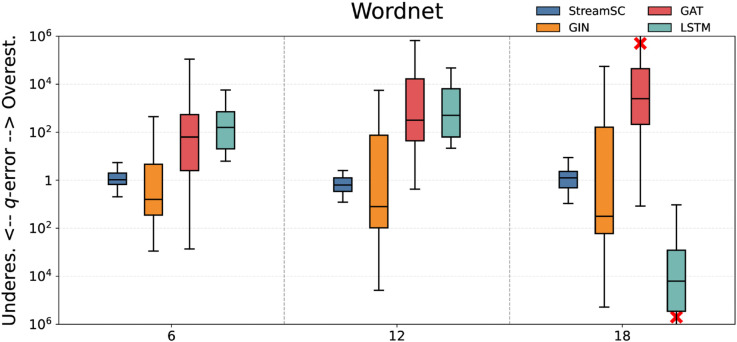
Subgraph counting results on Wordnet.

**Fig 8:**
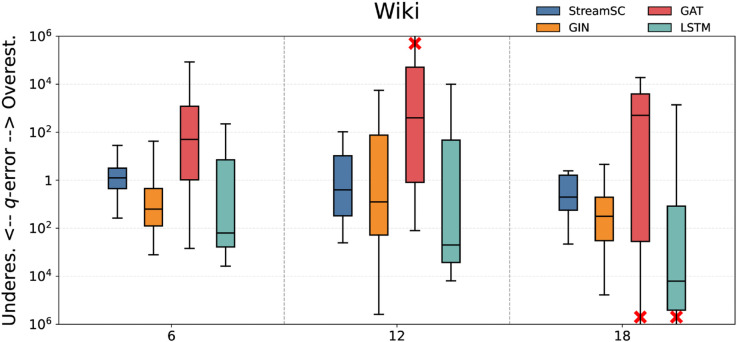
Subgraph counting results on Wiki.

**Fig 9:**
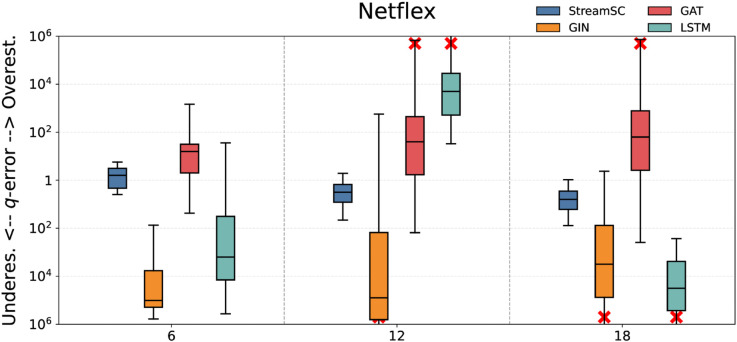
Subgraph counting results on Netflex.

We compared the predictive accuracy of StreamSC with baseline methods. On each dataset, we randomly generated three types of query sets with varying scales (node counts of 6, 12, and 18, respectively) to serve as test queries. The q-error values were calculated by comparing the predictions of each approximate method against the results obtained from the exact algorithm. The experimental results for all methods are illustrated in Figs 5–9.

As shown in the Figs 5–9, StreamSC effectively addresses the subgraph counting problem on stream graphs. Compared to classical GNNs methods, StreamSC achieves a lower q-error and exhibits a more concentrated distribution, demonstrating its superior stability across diverse query scenarios. This is because, compared to classical GNN methods, StreamSC can more effectively capture the mapping relationships between query graphs and data graphs, better leverage topological structures and maintain stable performance even when the data graph structure changes—thanks to its specialized design for stream graphs. These advantages are further validated by the MK test. The results of MK test are presented in Table 2.

**Table 2:** Performance comparison by dataset and node size.

Methods		StreamSC		GIN		GAT		LSTM	
Dataset	Nodes	$Z_{mk}$	*P*	$Z_{mk}$	*P*	$Z_{mk}$	*P*	$Z_{mk}$	*P*
Yeast	6	1.11	0.2684	2.63	0.0086	2.08	0.038	3.54	0.0004
	12	0.62	0.5326	2.15	0.0316	2.29	0.0221	2.21	0.0269
	18	1.80	0.0721	2.21	0.0268	2.35	0.0187	3.43	0.0006
Citeseer	6	1.33	0.1834	2.03	0.0423	2.14	0.0327	3.26	0.0011
	12	1.32	0.1877	3.81	0.0001	2.73	0.0063	1.59	0.1108
	18	0.49	0.6274	3.18	0.0015	2.15	0.0316	3.65	0.0003
WordNet	6	0.21	0.8325	2.39	0.0154	3.1	0.0019	2.91	0.0037
	12	1.25	0.2131	1.95	0.0516	3.18	0.0015	2.41	0.0159
	18	1.80	0.0726	2.49	0.0129	2.17	0.03	2.42	0.0151
Wiki	6	0.9	0.3704	2.43	0.0152	3.32	0.0009	2.21	0.0269
	12	1.81	0.0707	2.81	0.005	1.89	0.0587	3.32	0.01
	18	1.25	0.2131	3.24	0.0012	2.31	0.0209	3.99	0.0006
Netflex	6	1.52	0.1281	1.94	0.0526	3.54	0.0004	2.91	0.0036
	12	1.87	0.0612	3.18	0.0015	2.36	0.0182	3.13	0.0017
	18	1.53	0.1262	2.57	0.0103	2.01	0.0443	2.69	0.0072

P≥0.05 denotes no significant trend and smaller |*Z*_*mk*_| means more stable sequences.

As shown in Table 2, the experimental results demonstrate that StreamSC consistently achieves *p*-values above the significance threshold (α=0.05) across all datasets and query graph scales, indicating its ability to maintain stable prediction accuracy without significant error escalation as edges in the data graph dynamically change. In comparison, classical GNNs methods show limited stability - only GIN maintains stable performance for 6-node queries on Netflex and 12-node queries on Wordnet, while GAT exhibits similar stability solely for 12-node queries on Wiki. Notably, StreamSC achieves the smallest *p*-values in all test scenarios, reflecting its minimal error accumulation rate. These findings collectively establish StreamSC’s dual advantage of delivering both superior prediction accuracy and exceptional stability in stream graph environments, outperforming classical GNN approaches in handling evolving graph structures.

#### Efficiency of StreamSC.

We evaluate the efficiency of StreamSC by considering its end-to-end estimation time cost. As shown in Table 3, StreamSC has minimal time cost. We first take a close look at CSM. CSM represents the exact algorithm, which is a modification of continuous subgraph matching. It computes the exact subgraph counting results by performing subgraph matching. The time cost of CSM ranges from 107.14 seconds to 2331.39 seconds. In the Yeast dataset CSM exhibits the lowest time cost primarily because its pruning strategy leverages node types and neighborhood information to reduce the search space. Since the Yeast dataset has a relatively small graph size (fewer nodes and edges) and rich node-type diversity, making CSM’s pruning particularly effective. Conversely, CSM incurs the highest time cost on the Netflex dataset, mainly due to the large-scale nature of Netflex, which results in an expansive search space. Among approximation methods, StreamSC achieves orders-of-magnitude improvements in efficiency. This is because traditional methods require recomputing the entire data graph and query graph for every edge update whereas StreamSC’s specialized design eliminates these redundant computations, drastically reducing time cost. It is worth noting that we applied LearnSC to each snapshot of the stream graph to obtain its time overhead for processing the stream graph. While being the state of the art for subgraph counting on static graphs, LearnSC incurs the highest time overhead among all approximate methods—and even higher than exact methods. This is mainly due to its complex network architecture, which is designed to reduce prediction error. Furthermore, in the stream graph setting, LearnSC requires repeated loading and decomposition of the large data graph, which together result in its suboptimal performance. For this reason, we did not include it as a baseline in our study.

**Table 3:** End-to-end time cost of different methods.

Dataset	Nodes	Time Cost of Methods (s)					
		StreamSC	GIN	GAT	LSTM	CSM	LearnSC
Yeast	6	0.74	4.08	13.15	25.35	107.14	287.73
	12	0.77	6.12	15.22	22.48	107.51	412.83
	18	0.96	7.17	15.31	26.62	109.29	525.42
Citeseer	6	1.75	10.09	18.92	30.40	1184.73	1372.59
	12	1.21	10.49	19.49	32.55	1193.71	1227.63
	18	1.28	9.97	19.82	31.70	1187.43	1368.06
Wordnet	6	1.35	11.21	20.25	27.55	517.58	697.43
	12	1.07	10.98	20.36	26.65	520.12	711.81
	18	1.48	12.05	20.45	27.77	513.44	769.33
Wiki	6	0.81	10.26	19.35	27.50	1829.68	1903.68
	12	0.93	12.19	19.39	27.93	1821.38	2002.83
	18	1.02	12.25	19.95	27.02	1832.04	1844.19
Netflex	6	4.20	16.11	37.52	54.50	2309.56	2731.56
	12	4.31	16.16	37.61	55.53	2325.62	3079.70
	18	4.69	15.37	37.59	55.57	2331.39	2517.32

The time cost includes initial decomposition and the unit is seconds.

To further demonstrate the superiority of StreamSC, we evaluated its average response latency, which measures the average processing time per edge modification. As shown in Table 4, the local update mechanism in StreamSC effectively reduces redundant computations when the graph changes. Upon the arrival of an updated edge, StreamSC can promptly determine whether further computation is necessary. If so, it significantly narrows the scope of recomputation. Overall, these experiments provide strong evidence of StreamSC’s effectiveness in accelerating computation under dynamic updates.

**Table 4:** Average Response Latency of StreamSC.

Dataset	Nodes	Average response latency (ms)
Yeast	6	0.05
	12	0.05
	18	0.07
Citeseer	6	0.36
	12	0.24
	18	0.26
Wordnet	6	0.18
	12	0.14
	18	0.20
Wiki	6	0.04
	12	0.04
	18	0.05
Netflex	6	0.14
	12	0.15
	18	0.17

## 5 Discussion

Our experiments in Sect 4 comprehensively demonstrate the superiority of StreamSC in solving the subgraph counting problem on stream graphs. In terms of both prediction accuracy and stability under continuous edge updates, StreamSC outperforms conventional graph neural network (GNN) approaches. While classical GNNs methods exhibit significant prediction errors, our experimental results reveal that they can occasionally achieve relatively small errors, indicating their potential applicability to this problem. Indeed, existing GNNs-based methods for static graphs have demonstrated the capability to perform subgraph counting with small errors, which motivated our adoption of a learning-based approach in StreamSC. Notably, compared to static graphs, subgraph counting on stream graphs exhibits monotonicity properties that were not considered in existing static graph methods. By incorporating this characteristic into our loss function design, we further enhanced StreamSC’s performance. Regarding computational efficiency, our experiments clearly show that StreamSC significantly outperforms both exact algorithms and classical GNNs methods. While existing static graph subgraph counting methods can complete computations with reasonable time cost, they cannot be directly applied to stream graphs. The primary obstacle preventing the direct application of static graph approximation methods to streaming scenarios lies in the substantial redundant computations caused by frequent edge updates. To address this challenge, StreamSC employs two key algorithmic innovations: (1) performing re-computation only on the affected parts of data graph, and (2) enforcing model monotonicity through an innovative loss function. These optimizations effectively eliminate redundant computations and capture the monotonic properties of stream graphs, leading to substantial improvements in computational efficiency and estimation accuracy.

## Supporting information

S1 Appendix RAM cost. GPU acceleration was not used in our implementation, as our network model builds upon the LearnSC framework, which itself does not leverage GPUs. Consequently, we only present the peak memory consumption in Table.
(PDF)

S2 AppendixHyperparameter.Readers may be interested in how the hyperparameters $\lambda_{reg}$ and $\lambda_{mono}$ are selected. To provide insight, we conducted a simple analysis on the Yeast dataset, testing various combinations of these hyperparameters. We found that (0.3,0.7) yields the best performance. In the table, the reported average q-error performance for each combination is obtained by normalizing its mean q-error with respect to the mean q-error of the (0.3,0.7) setting. As shown in Table and in the experiments reported in the main text, no significant gradient explosion or vanishing was observed. This is attributed to the use of ReLU activations in the network, the application of tanh and normalization in the $L_{\text{mono}}$ term, and the logarithmic transformation of predicted and true values in the $L_{\text{reg}}$ term, as in LearnSC. Together, these measures keep both errors and gradients within a controlled range.(PDF)

S3 Appendix Average affected-substructure size. We report the average change in substructure size caused by edge updates.
(PDF)
