# Supplementary material for: Neural subgraph counting on stream graphs via localized updates and monotonic learning
Source: PLoS One. 2025 Oct 23;20(10):e0334724. doi: 10.1371/journal.pone.0334724 (PMC12548902; doi:10.1371/journal.pone.0334724)
Supplement: S1 Appendix — GPU acceleration was not used in our implementation, as our network model builds upon the LearnSC framework, which itself does not leverage GPUs. Consequently, we only present the peak memory consumption in Table. (PDF) [file pone.0334724.s001.pdf]

S1 Appendix. **RAM Cost.** GPU acceleration was not used in our implementation, as our network model builds upon the LearnSC framework, which itself does not leverage GPUs. Consequently, we only present the peak memory consumption in Table 1.

Table 1. Peak RAM Cost of Different Dataset

Datasets	Yeast	Citeseer	Wordnet	Wiki	Netflex
Peak RAM Cost(GB)	12	12	26	20	40
