# Supplementary material for: Neural subgraph counting on stream graphs via localized updates and monotonic learning
Source: PLoS One. 2025 Oct 23;20(10):e0334724. doi: 10.1371/journal.pone.0334724 (PMC12548902; doi:10.1371/journal.pone.0334724)
Supplement: S2 Appendix — Readers may be interested in how the hyperparameters λreg and λmono are selected. To provide insight, we conducted a simple analysis on the Yeast dataset, testing various combinations of these hyperparameters. We found that (0.3,0.7) yields the best performance. In the table, the reported average q-error performance for each combination is obtained by normalizing its mean q-error with respect to the mean q-error of the (0.3,0.7) setting. As shown in Table and in the experiments reported in the main text, no significant gradient explosion or vanishing was observed. This is attributed to the use of ReLU activations in the network, the application of tanh and normalization in the Lmono term, and the logarithmic transformation of predicted and true values in the Lreg term, as in LearnSC. Together, these measures keep both errors and gradients within a controlled range. (PDF) [file pone.0334724.s002.pdf]

S2 Appendix. Hyperparameter. Readers may be interested in how the hyperparameters λ_{reg} and λ_{mono} are selected. To provide insight, we conducted a simple analysis on the Yeast dataset, testing various combinations of these hyperparameters. We found that $(0.3, 0.7)$ yields the best performance. In the table, the reported average q-error performance for each combination is obtained by normalizing its mean q-error with respect to the mean q-error of the $(0.3, 0.7)$ setting.

Table 1. Different Hyperparameters Compared With $(0.3, 0.7)$

$(\lambda_{reg}, \lambda_{mono})$	(0.3,0.7)	(1,0)	(0.8,0.2)	(0.6,0.4)	(0.4,0.6)	(0.2,0.8)
q-error Performance	1	1.07	1.07	1.04	1.02	1.05

As shown in Table 1 and in the experiments reported in the main text, no significant gradient explosion or vanishing was observed. This is attributed to the use of ReLU activations in the network, the application of tanh and normalization in the L_{mono} term, and the logarithmic transformation of predicted and true values in the L_{reg} term, as in LearnSC. Together, these measures keep both errors and gradients within a controlled range.
