# Supplementary material for: Neural subgraph counting on stream graphs via localized updates and monotonic learning
Source: PLoS One. 2025 Oct 23;20(10):e0334724. doi: 10.1371/journal.pone.0334724 (PMC12548902; doi:10.1371/journal.pone.0334724)
Supplement: S3 Appendix — We report the average change in substructure size caused by edge updates. (PDF) [file pone.0334724.s003.pdf]

S3 Appendix. Average Affected-substructure Size. We report the average change in substructure size caused by edge updates.

Table 1. Average Number of Nodes in Affected-substructures

Dataset	Yeast	Citeseer	Wordnet	Wiki	Netflex
Average Number	34.7	246.2	173.8	48.2	149.1
